# Evaluation of SARS-CoV-2 RNA Rebound After Nirmatrelvir/Ritonavir Treatment in Randomized, Double-Blind, Placebo-Controlled Trials — United States and International Sites, 2021–2022

**DOI:** 10.15585/mmwr.mm7251a2

**Published:** 2023-12-22

**Authors:** Patrick R. Harrington, Jie Cong, Stephanie B. Troy, Jonathan M.O. Rawson, Julian J. O’Rear, Thamban Illath Valappil, Sarah McGarry Connelly, John Farley, Debra Birnkrant

**Affiliations:** ^1^Division of Antivirals, Office of Infectious Diseases, Center for Drug Evaluation and Research, Food and Drug Administration, Silver Spring, Maryland; ^2^Division of Biometrics IV, Office of Biostatistics, Center for Drug Evaluation and Research, Food and Drug Administration, Silver Spring, Maryland; ^3^Office of Infectious Diseases, Center for Drug Evaluation and Research, Food and Drug Administration, Silver Spring, Maryland.

**Summary**

**What is already known about this topic?**

Nirmatrelvir/ritonavir (Paxlovid) is recommended for treatment of mild-to-moderate COVID-19 in adults at high risk for progression to severe COVID-19. Rebound in SARS-CoV-2 shedding or COVID-19 signs and symptoms has been described after nirmatrelvir/ritonavir treatment, although the drug’s direct contribution to rebound remains unclear.

**What is added by this report?**

Similar SARS-CoV-2 RNA rebound rates were observed in nirmatrelvir/ritonavir and placebo recipients in two randomized, double-blind, clinical trials. Virologic rebound after nirmatrelvir/ritonavir treatment was not associated with COVID-19–related hospitalization or death.

**What are the implications for public health practice?**

SARS-CoV-2 RNA rebound can occur with or without nirmatrelvir/ritonavir treatment, supporting the Food and Drug Administration’s determination of safety and efficacy of nirmatrelvir/ritonavir in eligible patients at high risk for severe COVID-19.

## Abstract

Rebound of SARS-CoV-2 shedding or COVID-19 signs and symptoms has been described after treatment with nirmatrelvir/ritonavir (Paxlovid). The direct association of nirmatrelvir/ritonavir to COVID-19 rebound remains unclear because most reports are based on individual cases or nonrandomized studies. Viral RNA shedding data from two phase 2/3, randomized, double-blind, placebo-controlled clinical trials of nirmatrelvir/ritonavir (Evaluation of Protease Inhibition for COVID-19 in High-Risk Patients [EPIC-HR] and Evaluation of Protease Inhibition for COVID-19 in Standard-Risk Patients [EPIC-SR]) were analyzed to investigate the role of nirmatrelvir/ritonavir treatment in COVID-19 rebound. Rates of rebound of SARS-CoV-2 RNA shedding, identified based on an increase in nasopharyngeal viral RNA levels from day 5 (end-of-treatment) to day 10 or day 14, were similar between nirmatrelvir/ritonavir and placebo recipients. Among subjects with a virologic response through day 5, viral RNA rebound occurred in 6.4%–8.4% of nirmatrelvir/ritonavir recipients and 5.9%–6.5% of placebo recipients across EPIC-HR and the 2021/pre-Omicron and 2022/Omicron enrollment periods of EPIC-SR. Viral RNA rebound after nirmatrelvir/ritonavir treatment was not associated with COVID-19–related hospitalization or death. Data from randomized trials demonstrated that SARS-CoV-2 rebound can occur with or without antiviral treatment, supporting the Food and Drug Administration’s determination of safety and efficacy of nirmatrelvir/ritonavir in eligible patients at high risk for severe COVID-19.

## Introduction

Nirmatrelvir/ritonavir (Paxlovid)* is a COVID-19 oral antiviral treatment consisting of nirmatrelvir, a SARS-CoV-2 main protease (M^pro^ or 3C-like protease [3CL^pro^]) inhibitor, and ritonavir, a pharmacokinetic enhancer. Several reports have described patients who have experienced a rebound in SARS-CoV-2 detection or COVID-19 signs and symptoms after treatment with nirmatrelvir/ritonavir, or without antiviral treatment (*1*–*8*). Identifying the direct contribution of nirmatrelvir/ritonavir to the rebound phenomenon has been challenging. Other than limited analyses from the pharmaceutical sponsor of nirmatrelvir/ritonavir based on the Evaluation of Protease Inhibition for COVID-19 in High-Risk Patients (EPIC-HR) trial (*9*), conducted before SARS-CoV-2 Omicron emergence, reports of COVID-19 rebound are primarily based on individual cases and nonrandomized cohort studies. The lack of clarity and persistent concerns about COVID-19 rebound have reportedly contributed to reduced nirmatrelvir/ritonavir use (*10*). This analysis used data submitted to the Food and Drug Administration (FDA) to investigate the frequency of rebound in SARS-CoV-2 RNA shedding among outpatients with COVID-19 treated with nirmatrelvir/ritonavir or placebo in two randomized, double-blind clinical trials.

## Methods

### Data Sources

The authors conducted retrospective analyses of viral RNA shedding levels in nasopharyngeal samples from phase 2/3 clinical trials EPIC-HR and Evaluation of Protease Inhibition for COVID-19 in Standard-Risk Patients (EPIC-SR).^†^ Both were double-blind trials in which adult outpatients with mild-to-moderate COVID-19 were randomized 1:1 to receive 5 days of nirmatrelvir (300 mg)/ritonavir (100 mg) or placebo, twice daily. EPIC-HR enrolled participants in 2021, before the emergence of SARS-CoV-2 Omicron, and included adults who were unvaccinated against COVID-19 and at high risk for progression to severe disease. EPIC-SR originally enrolled two groups of participants in 2021, before Omicron emergence: 1) adults with high risk of SARS-CoV-2 exposure who had completed a primary vaccination series, and 2) unvaccinated adults without risk factors for severe disease. EPIC-SR reopened in 2022 when SARS-CoV-2 Omicron (primarily BA.2-related) predominated, and adults without risk factors for severe disease who had not received a COVID-19 vaccine during the preceding 12 months were enrolled.

Viral RNA levels were measured in nasopharyngeal swab samples collected by health care providers at prespecified study visits on days 1 (baseline), 3, 5 (end-of-treatment), 10, and 14 (Table 1).^§^ Viral RNA data from EPIC-HR used in these analyses overlap with those described in the 2022 study of nirmatrelvir/ritonavir and viral RNA rebound (*9*), although analysis definitions differed. 

**TABLE 1:** Definitions used to analyze SARS-CoV-2 RNA rebound in the Evaluation of Protease Inhibition for COVID-19 in High-Risk Patients and Evaluation of Protease Inhibition for COVID-19 in Standard-Risk Patients clinical trials — United States and international sites, 2021–2022

Analysis	Rationale	Parameters
Visit timepoints	SARS-CoV-2 RNA (log_10_ copies/mL) results from all protocol-specified study visits considered Visit windows based on protocol statistical analysis plans, one result per visit window	Day 1/Baseline (visit window: day −2 to 1), day 3 (day 2 to 4), day 5/End-of-treatment (day 4 to 6), day 10 (day 7 to 11), and day 14 (day 12 to 17) Results collected on day 4 assigned day 3 or day 5 based on planned study visit Day 1/Baseline = start of nirmatrelvir/ritonavir or placebo dosing
Posttreatment viral RNA rebound overall	Sensitive parameters to identify subjects with any evidence of an increase in SARS-CoV-2 RNA level in the posttreatment period	Day 10 rebound: day 5 RNA <LLOQ and day 10 RNA ≥LLOQ, or day 5 RNA ≥LLOQ and day 10 RNA ≥0.5 log_10_ copies/mL increase from day 5 Day 14 rebound: day 5 RNA <LLOQ and day 14 RNA ≥LLOQ, or day 5 RNA ≥LLOQ and day 14 RNA ≥0.5 log_10_ copies/mL increase from day 5 Day 10 or day 14 rebound: met either (or both) of the definitions above
Posttreatment viral RNA rebound among day 5 virologic responders	Subanalysis of posttreatment viral RNA rebound overall To account for greater impact of nirmatrelvir/ritonavir on viral RNA levels during treatment, and compare rebound rates between nirmatrelvir/ritonavir and placebo recipients with similar virologic responses through day 5	Day 5 virologic response: day 5 RNA <LLOQ, or ≥1 log_10_ copies/mL RNA decrease from baseline to day 5 and Day 10 or day 14 rebound (defined above)
Posttreatment viral RNA rebound with potentially infectious virus	Identification of posttreatment rebound with high viral RNA level at time of rebound potentially associated with cell culture infectivity	Day 10 or day 14 ≥5 log_10_ copies/mL rebound: day 10 or day 14 rebound (defined above), and day 10 or day 14 viral RNA ≥5 log_10_ copies/mL
Viral RNA rebound during treatment period	To identify nirmatrelvir/ritonavir or placebo recipients with a viral RNA response followed by rebound during the treatment period To compare rates of rebound in the treatment and posttreatment periods	Day 3 virologic response: day 3 RNA <LLOQ or ≥1 log_10_ copies/mL RNA decrease from baseline to day 3 and Day 3 to day 5 rebound: day 3 RNA <LLOQ and day 5 RNA ≥LLOQ, or day 3 RNA ≥LLOQ and day 5 RNA ≥0.5 log_10_ copies/mL increase from day 3
Cross-sectional viral RNA levels	To identify subjects with low viral RNA levels, or with high viral RNA levels potentially associated with cell culture infectivity, at individual timepoints irrespective of rebound	Viral RNA <LLOQ at indicated visit Viral RNA ≥5 log_10_ copies/mL at indicated visit

**Abbreviation:** LLOQ = lower limit of quantification.

### Data Analyses

Subjects with posttreatment viral RNA rebound were identified based on an increase in viral RNA levels from day 5 to day 10 or day 14 (Table 1). In a subanalysis to account for on-treatment virologic responses and assess more directly the impact of removing nirmatrelvir/ritonavir antiviral pressure after an initial viral RNA decline, rebound rates were compared between nirmatrelvir/ritonavir and placebo recipients who had a virologic response on day 5. To compare rebound rates in the treatment and posttreatment periods, viral RNA rebound rates during the treatment period between day 3 and day 5, among subjects with a virologic response on day 3, were determined. A viral RNA level ≥5 log_10_ copies/mL was considered potentially predictive of positive cell culture viral infectivity.^¶^

Exploratory statistical analyses were conducted using two-sided Fisher’s exact tests to provide nominal p-values, without multiplicity adjustments; p<0.05 was considered statistically significant. Analyses were conducted using JMP (version 16; SAS Institute). This study was reviewed by the FDA Institutional Review Board and deemed not to constitute research involving human subjects as defined in 45 CFR part 46.

## Results

### SARS-CoV-2 RNA Rebound Rates 

Demographic characteristics and predominant SARS-CoV-2 variants were similar between nirmatrelvir/ritonavir and placebo recipients within clinical trial EPIC-HR and within the 2021/pre-Omicron and 2022/Omicron periods of EPIC-SR (Supplementary Table, https://stacks.cdc.gov/view/cdc/136166). In EPIC-HR, overall rates of posttreatment viral RNA rebound on day 10 or day 14 were numerically higher in nirmatrelvir/ritonavir recipients than in placebo recipients, with most cases of observed rebound occurring at day 10 (Table 2). At either posttreatment timepoint (i.e., day 10 or day 14), viral RNA rebound rates in nirmatrelvir/ritonavir and placebo recipients were 8.3% (77 of 925) and 5.7% (53 of 922), respectively (p = 0.036). When the analysis was restricted to subjects with a virologic response on day 5, the difference between day 10 or day 14 rebound rates among nirmatrelvir/ritonavir and placebo recipients narrowed, and the rates were no longer significantly different (8.1% [69 of 849] and 6.5% [50 of 772], respectively; p = 0.22). In EPIC-SR, viral RNA rebound rates by either analysis approach were not significantly different between nirmatrelvir/ritonavir and placebo recipients across both enrollment periods.

**TABLE 2:** SARS-CoV-2 RNA rebound and responses in Evaluation of Protease Inhibition for COVID-19 in High-Risk Patients and Evaluation of Protease Inhibition for COVID-19 in Standard-Risk Patients clinical trials, by enrollment period — United States and international sites, 2021–2022

Responses	% (no./No.)		p*-*value*
	Nirmatrelvir/Ritonavir	Placebo	
**EPIC-HR, total no.^†^**	**1,038**	**1,053**	**—**
**Posttreatment viral RNA rebound**			
Day 10	6.6 (57/865)	4.7 (40/856)	0.095
Day 14	2.6 (23/884)	1.9 (17/893)	0.34
Day 10 or day 14	8.3 (77/925)	5.7 (53/922)	0.036
Day 10 or day 14 ≥5 log_10_ copies/mL	2.8 (26/925)	1.7 (16/922)	0.16
**Among day 5 virologic responders**			
Day 10 or day 14	8.1 (69/849)	6.5 (50/772)	0.22
Day 10 or day 14 ≥5 log_10_ copies/mL	2.6 (22/849)	1.9 (15/772)	0.41
**Viral RNA rebound during treatment period**			
Days 3−5^§^	11.3 (77/680)	12.5 (74/594)	0.54
**Viral RNA <LLOQ**			
Day 3	35.1 (340/970)	32.8 (321/980)	0.29
Day 5/End-of-treatment	47.8 (447/936)	44.1 (415/942)	0.12
Day 10	76.1 (702/922)	68.9 (622/903)	<0.001
Day 14	88.6 (835/942)	86.0 (815/948)	0.084
**Viral RNA ≥5 log_10_ copies/mL**			
Day 3	29.1 (282/970)	38.9 (381/980)	<0.001
Day 5/End-of-treatment	10.3 (96/936)	23.9 (225/942)	<0.001
Day 10	3.7 (34/922)	5.3 (48/903)	0.11
Day 14	1.0 (9/942)	1.5 (14/948)	0.40
**EPIC-SR 2021/pre-Omicron period, total no.**	**540**	**528**	**—**
**Posttreatment viral RNA rebound**			
Day 10	5.9 (28/477)	5.4 (25/467)	0.78
Day 14	2.4 (12/492)	1.5 (7/472)	0.36
Day 10 or day 14	6.6 (33/502)	6.2 (30/486)	0.90
Day 10 or day 14 ≥5 log_10_ copies/mL	3.4 (17/502)	1.9 (9/486)	0.16
**Among day 5 virologic responders**			
Day 10 or day 14	6.4 (31/482)	6.4 (27/421)	1.0
Day 10 or day 14 ≥5 log_10_ copies/mL	3.3 (16/482)	1.7 (7/421)	0.14
**Viral RNA <LLOQ**			
Day 3	31.5 (164/520)	30.3 (154/509)	0.69
Day 5/End-of-treatment	49.3 (251/509)	40.4 (199/492)	0.005
Day 10	77.3 (382/494)	72.1 (352/488)	0.066
Day 14	89.2 (456/511)	85.7 (425/496)	0.11
**Viral RNA ≥5 log_10_ copies/mL**			
Day 3	29.0 (151/520)	39.7 (202/509)	<0.001
Day 5/End-of-treatment	9.0 (46/509)	22.2 (109/492)	<0.001
Day 10	3.4 (17/494)	3.9 (19/488)	0.74
Day 14	1.8 (9/511)	1.4 (7/496)	0.80
**EPIC-SR 2022/Omicron period, total no.**	**114**	**106**	**—**
**Posttreatment viral RNA rebound**			
Day 10	6.0 (6/100)	4.0 (4/100)	0.75
Day 14	3.8 (4/105)	2.0 (2/102)	0.68
Day 10 or day 14	8.6 (9/105)	5.8 (6/103)	0.59
Day 10 or day 14 ≥5 log_10_ copies/mL	1.9 (2/105)	1.9 (2/103)	1.0
**Among day 5 virologic responders**			
Day 10 or day 14	8.4 (8/95)	5.9 (5/85)	0.57
Day 10 or day 14 ≥5 log_10_ copies/mL	1.1 (1/95)	2.4 (2/85)	0.60
**Viral RNA <LLOQ**			
Day 3	32.4 (35/108)	19.0 (20/105)	0.029
Day 5/End-of-treatment	56.6 (60/106)	36.5 (38/104)	0.004
Day 10	86.4 (89/103)	77.5 (79/102)	0.11
Day 14	91.7 (99/108)	90.4 (94/104)	0.81
**Viral RNA ≥5 log_10_ copies/mL**			
Day 3	38.0 (41/108)	52.4 (55/105)	0.04
Day 5/End-of-treatment	9.4 (10/106)	20.2 (21/104)	0.03
Day 10	1.9 (2/103)	2.9 (3/102)	0.68
Day 14	0.9 (1/108)	— (0/104)	1.0

**Abbreviations:** EPIC-HR = Evaluation of Protease Inhibition for COVID-19 in High-Risk Patients; EPIC-SR = Evaluation of Protease Inhibition for COVID-19 in Standard-Risk Patients; LLOQ = lower limit of quantification.

* Nominal p-values shown based on two‐sided Fisher’s exact test, unadjusted for multiplicity.

^†^ Total number of randomized subjects who took ≥1 dose of study intervention and had initial onset of COVID‐19 signs and symptoms within 5 days of randomization. Denominators for viral RNA rebound and response rates are based on the number of subjects with data at the relevant visit timepoints.

^§^ Assessed in subjects with a virologic response on day 3.

For both trials, when considering a higher threshold for viral RNA rebound (requiring a day 10 or day 14 viral RNA ≥5 log_10_ copies/mL, associated with cell culture infectivity) viral RNA rebound rates remained similar for nirmatrelvir/ritonavir and placebo recipients. Further, no consistent differences in viral RNA patterns were observed between nirmatrelvir/ritonavir and placebo recipients with viral RNA rebound (Supplementary Figure, https://stacks.cdc.gov/view/cdc/136167). In the analysis of EPIC-HR data, viral RNA rebound during the treatment period was frequently observed, with rates numerically higher than posttreatment rebound rates (Table 2).

### Hospitalization, Immunosuppression, and Antiviral Resistance Among Study Subjects

Viral RNA rebound was generally not associated with COVID-19–related hospitalization or death from any cause through day 28. Among subjects in EPIC-HR with viral RNA rebound, 1.3% (one of 77) of nirmatrelvir/ritonavir recipients and 5.7% (three of 53) of placebo recipients had a COVID-19–related hospitalization, without death, comparable with overall hospitalization rates in the trial.** In EPIC-SR, three subjects (one nirmatrelvir/ritonavir and two placebo recipients; 2021/pre-Omicron period) had viral RNA rebound and a COVID-19–related hospitalization. Analyses of viral RNA levels showed no consistent temporal relationship between viral RNA rebound and hospitalization (Figure). Viral RNA rebound was not associated with immunosuppression; however, only 13 subjects in EPIC-HR were immunosuppressed, including six nirmatrelvir/ritonavir recipients (none with rebound) and seven placebo recipients (one with rebound).

**FIGURE:**
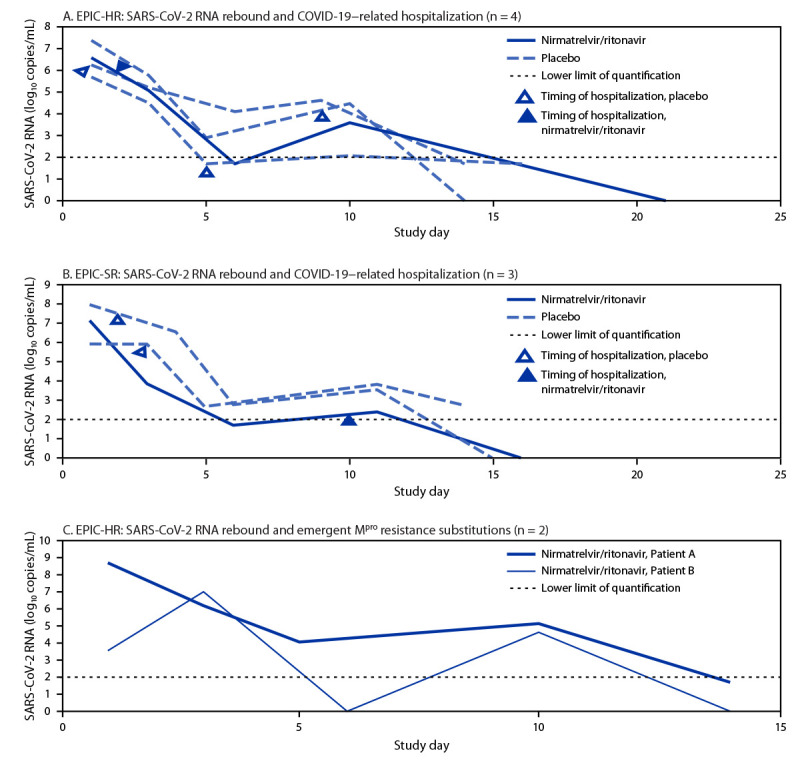
SARS-CoV-2 RNA shedding levels for subjects with viral RNA rebound who experienced COVID-19–related hospitalization any time through day 28 in the Evaluation of Protease Inhibition for COVID-19 in High-Risk Patients (A) and Evaluation of Protease Inhibition for COVID-19 in Standard-Risk Patients (B) clinical trials, and two subjects with evidence of treatment-emergent nirmatrelvir resistance–associated substitutions detected in the viral main protease gene (C)* — United States and international sites, 2021–2022 **Abbreviations:** EPIC-HR = Evaluation of Protease Inhibition for COVID-19 in High-Risk Patients; EPIC-SR = Evaluation of Protease Inhibition for COVID-19 in Standard-Risk Patients; M^pro^ = viral main protease gene. * On study day 10, 23.9% of patient A’s M^pro^ sequence reads had a T304I substitution, and 23.6% of patient B’s M^pro^ sequence reads had an E166V substitution.

Among 59 nirmatrelvir/ritonavir recipients in EPIC-HR with viral RNA rebound for whom viral sequencing data were available, two (3%) had a treatment-emergent, nirmatrelvir resistance–associated substitution detected in M^pro^ on day 10 (Figure). Neither subject was immunosuppressed at baseline or hospitalized because of COVID-19.

### SARS-CoV-2 RNA Levels at Individual Timepoints 

Across both trials, for all subjects irrespective of viral RNA rebound by any definition, a similar or higher percentage of nirmatrelvir/ritonavir recipients than placebo recipients had viral RNA below the lower limit of quantification (LLOQ) at all postbaseline visits, indicating nirmatrelvir/ritonavir treatment was not associated with delayed viral clearance overall (Table 2). Likewise, a similar or lower percentage of nirmatrelvir/ritonavir recipients than placebo recipients had viral RNA ≥5 log_10_ copies/mL at all postbaseline visits.

## Discussion

Analyses of nasopharyngeal SARS-CoV-2 RNA levels from two randomized, double-blind, placebo-controlled trials that collectively enrolled approximately 3,000 subjects did not identify a consistent association between virologic rebound and nirmatrelvir/ritonavir treatment. One analysis from EPIC-HR indicated a statistically significantly higher rate of viral RNA rebound overall in nirmatrelvir/ritonavir recipients compared with that in placebo recipients (8.3% versus 5.7%, respectively; p = 0.036), but this analysis did not account for differences in viral RNA declines while on treatment. Other analyses from EPIC-HR and EPIC-SR did not show significant differences but did show modestly (nonsignificant) higher viral RNA rebound rates in nirmatrelvir/ritonavir recipients. Collectively, these data indicate that viral RNA rebound might be more common with nirmatrelvir/ritonavir treatment. However, viral RNA rebound was not restricted to nirmatrelvir/ritonavir recipients, and rebound rates were generally similar to those in placebo recipients across all analyses. Further, regardless of virologic rebound, nirmatrelvir/ritonavir treatment did not appear to contribute to delayed viral clearance overall, as nirmatrelvir/ritonavir recipients were more likely than were placebo recipients to have viral RNA levels below the LLOQ at all study visits. Viral RNA rebound during the treatment period between day 3 and day 5 was frequently observed, indicating that viral RNA rebound after treatment cannot definitively be attributed to virologic relapse caused by drug clearance and loss of antiviral activity. Rather, at least some cases of posttreatment rebound likely reflect natural variability in virus production, periods of shedding of viral components related to host factors, or technical variability in sampling via topical swab, any of which might also explain the occurrence of rebound in placebo recipients. Although nirmatrelvir drug resistance was not typically associated with viral RNA rebound, consistent with previous studies, two nirmatrelvir/ritonavir-treated subjects in EPIC-HR had virus with nirmatrelvir resistance-associated substitutions at the time of rebound. Genomic databases should continue to be monitored for the emergence or spread of nirmatrelvir-resistant SARS-CoV-2 variants.

### Limitations

The findings in this report are subject to at least four limitations. First, rebound rates are highly dependent on analysis definitions and the types, frequency, and timing of sample collection. The described analyses used sensitive parameters (within limitations of available sampling timepoints) to identify viral RNA rebound, and unlike a previous analysis from EPIC-HR (*9*), rebound only at a single posttreatment timepoint was sufficient to identify subjects as having viral RNA rebound. However, some rebound events were likely missed between study visits or after day 14. Given that viral RNA rebound rates in nirmatrelvir/ritonavir and placebo recipients were similar and progressively declined on days 3–5, 10, and 14 (and further considering the short half-life of nirmatrelvir), a significant association between nirmatrelvir/ritonavir and rebound only when considering intermediate timepoints or only after day 14 is considered unlikely, although this possibility cannot be excluded. Second, EPIC-HR included only 13 subjects with immunosuppression, in whom posttreatment COVID-19 rebound might be more common or clinically significant. Third, subjects at high risk infected with SARS-CoV-2 Omicron or subsequent sublineages were not represented because of the availability of nirmatrelvir/ritonavir through Emergency Use Authorization after Omicron emergence. Nevertheless, the analyses identified both nirmatrelvir/ritonavir and placebo recipients with viral RNA rebound within the EPIC-SR 2022/Omicron population. Finally, analyses focused on objective virologic measures and severe disease outcomes as reflected by COVID-19–related hospitalization or death, and available data did not permit a detailed investigation into less severe disease signs and symptoms.

### Implications for Public Health Practice

Data from randomized, double-blind clinical trials demonstrated similar rates of SARS-CoV-2 RNA rebound in nirmatrelvir/ritonavir and placebo recipients. These findings support FDA’s determination of safety and efficacy of nirmatrelvir/ritonavir in eligible patients at high risk for severe COVID-19.

## Footnotes

* Nirmatrelvir/ritonavir (Paxlovid) first became available in the United States for the treatment of mild-to-moderate COVID-19 through Emergency Use Authorization from the Food and Drug Administration (FDA) on December 22, 2021, (https://www.fda.gov/media/155049/download) and was approved by FDA on May 25, 2023, for the treatment of mild-to-moderate COVID-19 in adults who are at high risk for progression to severe COVID-19, including hospitalization or death. https://www.accessdata.fda.gov/drugsatfda_docs/appletter/2023/217188Orig1s000ltr.pdf

† Data from clinical trials EPIC-HR and EPIC-SR were submitted to FDA by the applicant (Pfizer) in support of the review and approval of New Drug Application (NDA) 217188 (Paxlovid). Analyses of viral RNA and symptom rebound for this report were conducted for all randomized subjects in these trials who took ≥1 dose of nirmatrelvir/ritonavir or placebo and were randomized ≤5 days of symptom onset. https://www.accessdata.fda.gov/drugsatfda_docs/nda/2023/217188Orig1s000IntegratedR.pdf

§ Viral RNA levels in nasopharyngeal samples were measured in a central laboratory (University of Washington) using the Abbott RealTime Quantitative SARS-CoV-2 assay, which had a lower limit of quantification (LLOQ) and limit of detection of 2 log_10_ copies/mL. For analysis purposes, SARS-CoV-2 RNA results that were detected, but <LLOQ were imputed as 1.7 log_10_ copies/mL, and the results that were “Not Detected” were imputed as zero log_10_ copies/mL. In limited cases (4%–6% of visits), samples were not explicitly reported as nasopharyngeal swabs (e.g., nasal swabs).

¶ Analysis of selected samples from EPIC-HR for cell culture infectious SARS-CoV-2 demonstrated a trend of positive infectivity for samples with viral RNA ≥5 log_10_ copies/mL (per FDA Integrated Review of NDA 217188). https://www.accessdata.fda.gov/drugsatfda_docs/nda/2023/217188Orig1s000IntegratedR.pdf

** In the EPIC-HR modified intent-to-treat-2 population, which included all randomized subjects who took ≥1 dose of study intervention and were dosed ≤5 days of symptom onset, 1.0% (10 of 1,038) of nirmatrelvir/ritonavir recipients and 6.2% (65 of 1,053) of placebo recipients experienced COVID-19–related hospitalization through day 28 (per FDA Integrated Review of NDA 217188). https://www.accessdata.fda.gov/drugsatfda_docs/nda/2023/217188Orig1s000IntegratedR.pdf
